# Local Differences in Cortical Excitability – A Systematic Mapping Study of the TMS-Evoked N100 Component

**DOI:** 10.3389/fnins.2021.623692

**Published:** 2021-02-25

**Authors:** Daniela Roos, Lea Biermann, Tomasz A. Jarczok, Stephan Bender

**Affiliations:** Department of Child and Adolescent Psychiatry, Psychosomatics, and Psychotherapy, Faculty of Medicine and University Hospital Cologne, University of Cologne, Cologne, Germany

**Keywords:** N100, TMS-EEG study, motor cortex mapping, cortical excitability, relationship N100 and MEP

## Abstract

Transcranial magnetic stimulation (TMS) with simultaneous electroencephalography applied to the primary motor cortex provides two parameters for cortical excitability: motor evoked potentials (MEPs) and TMS-evoked potentials (TEPs). This study aimed to evaluate the effects of systematic coil shifts on both the TEP N100 component and MEPs in addition to the relationship between both parameters. In 12 healthy adults, the center of a standardized grid was fixed above the hot spot of the target muscle of the left primary motor cortex. Twelve additional positions were arranged in a quadratic grid with positions between 5 and 10 mm from the hot spot. At each of the 13 positions, TMS single pulses were applied. The topographical maximum of the resulting N100 was located ipsilateral and slightly posterior to the stimulation site. A source analysis revealed an equivalent dipole localized more deeply than standard motor cortex coordinates that could not be explained by a single seeded primary motor cortex dipole. The N100 topography might not only reflect primary motor cortex activation, but also sum activation of the surrounding cortex. N100 amplitude and latency decreased significantly during stimulation anterior-medial to the hot spot although MEP amplitudes were smaller at all other stimulation sites. Therefore, N100 amplitudes might be suitable for detecting differences in local cortical excitability. The N100 topography, with its maximum located posterior to the stimulation site, possibly depends on both anatomical characteristics of the stimulated cortex and differences in local excitability of surrounding cortical areas. The less excitable anterior cortex might contribute to a more posterior maximum. There was no correlation between N100 and MEP amplitudes, but a single-trial analysis revealed a trend toward larger N100 amplitudes in trials with larger MEPs. Thus, functionally efficient cortical excitation might increase the probability of higher N100 amplitudes, but TEPs are also generated in the absence of MEPs.

## Introduction

Transcranial magnetic stimulation (TMS) is a noninvasive technique to examine excitability in the brain. Many TMS studies target primary motor cortex (M1) because changes in the excitability of the cortical motor neurons can be quantified by motor evoked potentials (MEPs). However, MEPs only deliver indirect information about cortical activation as they are susceptible to noncortical confounds, such as spinal cord excitability (Chung et al., 2015). In contrast, TMS combined with electroencephalography (EEG) enables direct measurement of neuronal responses and reveals specific information about the excitability of cortical areas (Hallett, 2007; Komssi et al., 2007; Ilmoniemi and Kičić, 2010). This approach records neuronal responses to TMS in the EEG as TMS-evoked potentials (TEP). The N100 is one of the most studied TEP components and is characterized by a negative peak at a latency of approximately 100 ms after the TMS pulse (Nikulin et al., 2003; Komssi et al., 2004; Bender et al., 2005). The N100 is considered a stable indicator of cortical inhibition, which is modulated through the activity of the inhibitory neurotransmitter GABA-B, causing the characteristic long N100 latency (Nikulin et al., 2003; Premoli et al., 2014). A recent study further suggests that N100 amplitude reflects not only local activity of GABA, but a balance between concentrations of GABA and excitatory glutamate (Du et al., 2018). N100 might, therefore, be a marker for cortical excitability in general. Additionally, the N100 has been evoked in various cortical areas (e.g., motor cortex, prefrontal cortex, and parietal cortex; Lioumis et al., 2009; Casarotto et al., 2010; Kerwin et al., 2018), suggesting that it is generated by local stimulated cortex with topographical maxima ipsilateral to the stimulation site (Bender et al., 2005; Bonato et al., 2006). Although these findings suggest the N100 is an indicator of local excitability, Du et al. (2017) suggest that the N100 instead reflects a global neuronal response because stimulation of various cortical areas leads to similar N100 topographies at the vertex. An important next step is to, therefore, investigate the cortical generators of TEPs and, in particular, how stimulation position affects topography and amplitude of the N100 component. These questions can be optimally addressed by applying TMS-EEG within the motor cortex, allowing MEPs to provide additional information on the magnitude of motor cortex stimulation (Julkunen et al., 2009).

Two approaches may clarify the topography and cortical origin of the N100: analysis of coil positioning effects on N100 topography and source analysis of multichannel TEP. If the N100 reflects local cortical excitability, amplitudes should covary with changes to the stimulation position around the motor cortex hot spot. One previous study compared TEP deflections across nine different stimulation targets, located 2 and 5 mm around the target muscle hot spot (de Goede et al., 2018), and observed no group-level differences in TEP deflections at a latency of 100 ms. In our study, we aim to assess larger variation in coil positioning (5–10 mm) to test whether larger distances between coil positions reveal changes in local cortical excitability and N100 topography. To our knowledge, these two aspects have not yet been investigated. Furthermore, source analysis should demonstrate whether the N100 TEP component resulting from focal activation of primary motor cortex can be sufficiently explained by a single equivalent dipole near the cortical surface with radially (crown of the precentral gyrus) and tangentially oriented components (posterior wall of the precentral gyrus) (Bruckmann et al., 2012).

Although TEPs and MEPs both occur following motor cortex stimulation, the relationship between both parameters is not yet fully understood. Although previous studies report heterogeneous results on correlations (Paus et al., 2001; Bender et al., 2005), higher TEP amplitudes are associated with higher MEP amplitudes at the intraindividual level (Fecchio et al., 2017). Comparing the effects of stimulation position within the motor cortex between the N100 and MEPs would provide further information about the relationship between these two parameters (i.e., do both the N100 and MEP amplitudes decline with a similar gradient as the coil moves away from the motor hot spot?).

Our study assesses the topography and cortical origin of the N100 by measuring the effects of systematic changes in stimulation position on both N100 and MEP amplitudes. We test the hypothesis that TMS leads to a topographical maximum of the N100 ipsilateral to stimulation position. We predict that the topographic maximum of the N100 is located at the respective stimulation site, i.e., covaries with changes in coil position. We further predict that N100 amplitudes are similar in magnitude following stimulation at different positions given that cortical excitability could be equal across stimulation positions, consistent with the results of de Goede et al. (2018). Our second hypothesis concerns the cortical origin of the N100; we expect that primary motor cortex activation can be represented by a single equivalent dipole in the motor cortex. Our third hypothesis is guided by findings that MEP amplitudes can only be measured in small areas in the primary motor cortex (Herwig et al., 2001, 2002) and are highest above the motor hot spot (Julkunen et al., 2009). Accordingly, we expect that MEP amplitudes are highest over the motor hot spot and decrease as the stimulation location departs this site. Due to the different predicted effects of stimulation location on MEP and N100 amplitudes, we do not expect to find a correlation between stimulation location–driven amplitude changes in the N100 and those in MEPs.

## Materials and Methods

This study was approved by the ethics committee of the Faculty of Medicine, University of Cologne, Germany (application number 17/305), and all procedures were performed in accordance with the Declaration of Helsinki. Data were collected exclusively for this study and reported here for the first time. Data cannot be shared as the local ethics committee did not give permission to share data online.

### Subjects

Fifteen healthy, right-handed adults participated in the study (handedness was assessed by the Edinburgh Handedness Inventory; Oldfield, 1971). No reliable motor hot spot could be determined in three participants, and they were excluded from further analysis. For all other subjects, the highest MEP interpeak amplitudes occurred consistently during stimulation of the motor cortex hot spot (see Supplementary Table A.1 for group statistics). These 12 analyzed subjects (9 women; mean age 23.4 ± 2.3 years) had no neurological or psychiatric diseases and were all free of medication. Participants gave informed written consent and obtained monetary compensation for their participation. TMS safety guidelines and TMS exclusion criteria for healthy subjects were taken into account (Rossi et al., 2011).

### TMS

**FIGURE 1:**
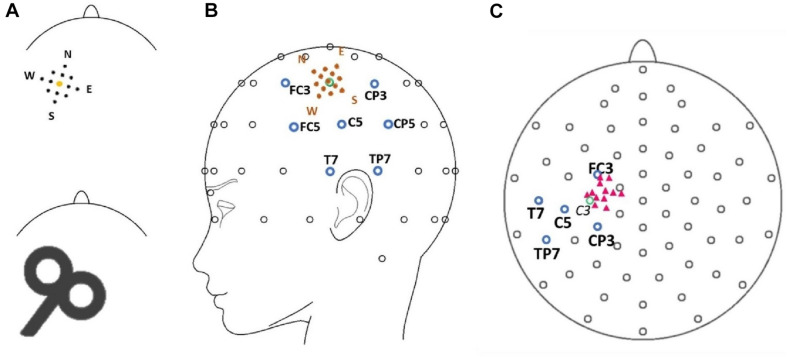
**(A)** Model of the mapping grid with the corners of the grid named after cardinal points (North, East, South, West) (upper row) as well as a scheme of the stimulation coil (lower row) pictured with the approximate alignment in relation to the shape of the head (view is from the top of the head with the nose pointing upward). Sizes are not true to scale. **(B)** The position of the grid (orange dots) is illustrated in spatial relation to the electrode montage. Blue circles mark the electrode in the center of the topographical maximum (C5) and neighboring electrodes (FC3, CP3, TP7, T7, CP5, FC5). The green circle marks the position of electrode C3. **(C)** Pink triangles mark the approximate positions of the individual target muscle motor hot spots during the experimental procedure in relation to the 10–20 electrode system of the 12 participants. The green circle marks the position of electrode C3. Note that the projection (top view) distorts the distances between electrodes.

A biphasic, single-pulse TMS protocol was applied with a handheld figure-of-eight coil (MCF-B65, outer diameter 2X 75 mm) connected to a MagPro X100 with MagOption stimulator (MagVenture, Farum, Denmark). For all subjects, the left primary motor cortex hot spot for the right first dorsal interosseous (FDI) was identified by positioning the coil’s center over the left primary motor cortex tangentially at a 45° angle from the midsagittal line with the handle facing backward. The hot spot was defined as the site with a reliable MEP interpeak amplitude > 50 μV (Rossini et al., 2015, 1994). Resting motor thresholds (RMT) were determined with a maximum likelihood procedure (Awiszus and Borckardt, 2006), using the Motor Threshold Assessment Tool (MTAT, version 2.0^1^). Stimulation intensity was set to 120% RMT for all experimental data acquisition. Individual hot spots served as the sites for the central position of the stimulation grid and were located approximately anterior and medial from electrode C3 in the direction of electrodes FC3 and FC1 (Figure 1). In previous studies, the locations of the primary motor cortex and the hot spot for the FDI muscle were associated with electrode C3 in the 10–20 electrode system (Rogasch et al., 2013; Holmes and Tamè, 2019).

### Experimental Protocol

All participants were seated in an armchair with their elbows flexed at a 90° angle, hands pronated in a relaxed position with eyes open. Participants were asked to visually fixate on a cross 1 m in front of them to reduce eye movement. The motor hot spot was marked with a cross on the electrode cap. A mapping grid, centered over the hot spot, was fixed on the electrode cap as well. The mapping grid consisted of 12 positions around the hot spot. The stimulation sites were arranged in a 20 × 20 mm square with four sites 10 mm, four sites approximately 7 mm, and four sites 5 mm from the hot spot. The stimulation sites were named using the following convention: “north” (N) 10 mm anterior-medially, “east” (E) 10 mm posterior-medially, “south” (S) 10 mm posterior-laterally, and west (W) 10 mm anterior-laterally to the hot spot. The motor hot spot stimulation at the center of the grid was termed “central” (C). The eight sub-positions in between the above main points had compound names comprised of the two closest main stimulation sites—i.e., “north-central” (NC, 5 mm to hot spot), “north-east” (NE, 7 mm to hot spot), “north-west” (NW, 7 mm to hot spot), “east-central” (EC, 5 mm to hot spot), “south-central” (SC, 5 mm to hot spot), “south-east” (SE, 7 mm to hot spot), “south-west” (SW, 7 mm to hot spot), and “west-central” (WC, 5 mm to hot spot) (see Figure 1). Forty-five TMS single pulses were applied at every position of the mapping grid with stimulus intervals varying between 5 and 8 s. The order of the stimulated sites was counterbalanced (e.g., 45 trials at NC then 45 trials at S, etc.).

### Data Recording

Brain and muscle activity was simultaneously recorded using an EEG and an electromyogram (EMG), respectively. EEG data were recorded at a sampling rate of 5000 Hz with a 64-channel BrainAmp system (BrainProducts, Munich, Germany) using Brain Vision Recorder software (BrainProducts). EEG data were recorded with a TMS-compatible EEG cap (Easycap, Inning am Ammersee, Germany) with electrodes placed in an equidistant montage similar to the extended 10–10 system with additional electrooculogram electrodes under the right and left eyes and on the nasion. Cz served as the recording reference, and the ground electrode was placed near Pz. Impedances for all electrodes were kept below 5 kΩ. EMG data were recorded with two self-adhesive silver–silver chloride electrodes placed in a belly tendon montage over the right FDI with a ground electrode placed on the inner forearm.

### Data Preprocessing

Both EEG and EMG data were preprocessed and analyzed using BrainVision Analyzer software (BrainProducts, Munich, Germany). Due to their large file sizes, EEG recordings were down-sampled to 500 Hz. Although the implemented antialiasing filter (low-pass filter 225 Hz) in the down-sampling process led to a slight distortion of the TMS pulse artifact (Rogasch et al., 2017), the introduced ringing artifact affected only early latencies below 20–30 ms and not late components, such as the N100. To remove the high-amplitude TMS pulse artifact, time segments from -10 to 20 ms around the TMS pulse were edited using linear interpolation. EEG data were re-referenced to the montage average and segmented into epochs from −500 to 500 ms around the TMS pulse. Severe remaining artifacts (e.g., large muscular artifacts) were rejected manually by visual inspection (less than 4% of the data in individual channel mode was rejected). Independent component analysis was performed on each subject, and components related to blink and eye-movement artifacts were removed (Ilmoniemi et al., 2015). The baseline was set from −110 to −10 ms prior to the TMS pulse (excluding the final 10 ms pre-pulse to avoid baseline contamination by the high-amplitude TMS artifact). Linear DC detrending was applied to all EEG channels. We additionally compared results using data with and without DC detrend to ensure that DC detrending did not have any systematic effects on the N100 deflection. Data were averaged separately for all experimental conditions (i.e., each stimulation site). The EMG signal was high-pass filtered at 20 Hz and averaged for all experimental conditions as well.

### Data Analysis

#### N100

**FIGURE 2:**
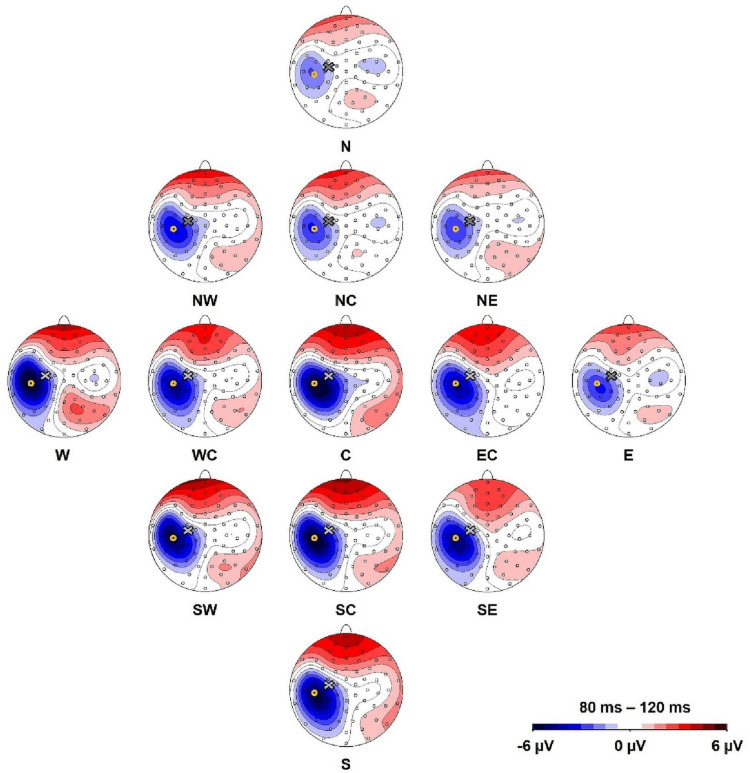
Topography of the N100 component for all stimulation conditions (North, North-West, North-East, North-Central, Central, East-Central, East, South-East, South, South-Central, South-West, West, West-Central). Yellow circles mark the position of electrode C5 and the cross marks the approximate TMS stimulation site during hot spot stimulation (group average). The view is from the top of the head with the nose pointing upward.

We defined a region of interest for data analysis and selected the electrode of interest based on the location of the most distinctive TMS-evoked N100 response (Ilmoniemi and Kičić, 2010). Here, we preempt parts of the later results to describe this process. The topographical maximum of the N100 in the group average in all stimulation conditions was located slightly posterior to the motor hot spot locations (Figure 2). The highest mean N100 amplitude during hot spot stimulation was recorded at electrode C5 (see Supplementary Table A.1). This topographical maximum is in line with previous findings that report widely distributed N100 topographies posterior to C3 (Paus et al., 2001; Bonato et al., 2006; Bruckmann et al., 2012; Määttä et al., 2017; Kaarre et al., 2018). We, therefore, used C5 and defined the N100 peak as the most negative peak recorded from this channel in the time window from 80 to 120 ms following each stimulation condition for all single-subject averages. Time windows spanning ± 10 ms around these peaks were exported for statistical analysis. In single-subject averages with positive N100 values, N100 amplitudes were set to zero to fulfill the definition criteria of a negative peak and to reduce the impact of outliers. This was only necessary in 8.8% of all analyzed single-subject averages. When positive N100 values were also included in the analysis, results did not change.

**FIGURE 3:**
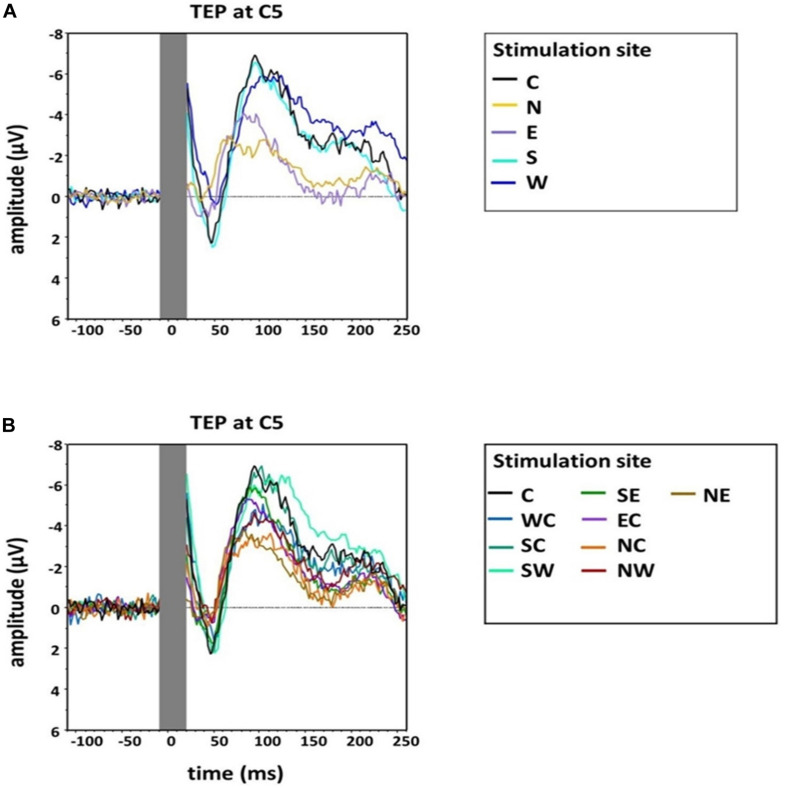
Group grand average of N100 amplitudes at electrode C5 for **(A)** the main stimulation positions (Central, North, West, South, East) and **(B)** sub-directions with the central position as comparison (Central, North-East, North-Central, North-West, West-Central, South-West, South-Central, South-East, East-Central). The TMS artifact (gray bar) has been cut out.

An exploratory visual analysis of the N100 waveform in grand averages revealed that the N condition contained two peaks between 80 and 120 ms poststimulation (Figure 3). We accordingly conducted a second peak detection for all main conditions (i.e., C, N, E, S, and W) using a window from 80 to 100 ms poststimulation, which controls for the confounding effect of the second peak in the N condition when using the original time window.

#### Global Field Power

Global field power (GFP) over all electrodes was calculated (Lehmann and Skrandies, 1980; Skrandies, 1990) separately per condition to test whether overall cortical activity changed as a function of coil position. Mean voltage in the time window 80–120 ms was exported for statistical analysis.

#### Source Analysis

We investigated the cortical origin of the N100 with a spatiotemporal source analysis implemented in BESA 7.1 (Munich, Germany). To check the degree to which primary motor cortex activation explained N100 topography, we fitted a single dipole to the N100 in single-subject averages (time interval 80–120 ms) for the hot spot (C) stimulation condition. We maximized explained variance using the generic algorithm implemented in BESA and used a four-sphere head model. As the hot spot condition topography showed only one prominent topographic peak, no further dipoles were necessary, i.e., to account for other evoked potential components or artifacts. As a complementary source analysis, we also employed low-resolution electromagnetic tomography (LORETA in BESA 7.1) using distributed sources both with and without a limitation of the source space to the cortex.

#### MEP

Motor evoked potentials interpeak amplitudes were determined manually and exported for statistical analysis. We calculated the percentage change in MEP interpeak amplitudes as well as in N100 amplitudes recorded during the four main stimulation conditions (N, E, S, W), relative to the hot spot (C). Furthermore, in the main stimulation conditions (C, N, E, S, W) trials with “high” MEP amplitudes (rectified amplitude > 20 μV) and trials with “low” MEP deflections were separately averaged to compare N100 amplitudes between both MEP groups.

### Statistics

Statistical analyses were performed using IBM SPSS Statistics 25 software (IBM Corp., Armonk, NY, United States). Due to the nonnormal distribution of N100 amplitudes (Shapiro–Wilk test *p* < 0.05), we tested experimental effects using nonparametric one-way Friedman’s ANOVAs with two-tailed asymptotic significances. Post hoc tests were conducted with Wilcoxon signed rank tests for two related samples with two-tailed exact significance and were Bonferroni corrected. A statistical significance level of *p <* 0.05 was used. Effect sizes were estimated using a *z*-score normalized by sample size, i.e., $r=z/\sqrt{N}$(Rosenthal, 1991). We summarize data using median and interquartile range (IQR: 25%–75%).

#### N100 Topography

Exploratory visual analyses of both grand and individual averages for the four main conditions (N, E, S, W) were performed to check for small systematic shifts in the N100 topography due to coil positioning. We then tested statistically whether N100 amplitudes changed relative to the site of peak amplitude (C5) at neighboring electrode sites. The closest neighboring electrodes to C5 were FC5 (anterior/N), CP3 (medial/E), CP5 (posterior/S), and T7 (lateral/W). Due to the angle of the grid, electrodes FC3 (anterior-medial to C5) and TP7 (posterior-lateral to C5) could also represent the coil positions N and S despite being located further away from C5 than electrodes FC5 and CP5 (Figure 1). We, therefore, considered the relative amplitudes of FC5/CP5 for anterior versus posterior topography shifts (N–S axis), FC3/TP7 for anterior-medial versus posterior-lateral topography shifts (second N–S axis), and T7/CP3 for lateral to medial topography shifts (E–W axis). To determine the relative amplitudes, a quotient was calculated between the N100 amplitudes of the two electrodes of each axis for the respective main stimulation conditions (e.g., N100 amplitude at FC3 during stimulation N/N100 amplitude at TP7 during stimulation N). The two quotients per axis were compared with each other with two-tailed Wilcoxon signed rank tests. Single electrodes were used instead of regions of interest because we expected the small variation in coil location to drive small topography changes that may have been lost in averages across multiple electrode sites. Finally, we checked that results did not differ when FC5 and CP5 were examined instead of FC3 and TP7.

#### Source Analysis

To assess whether N100 topography could be explained by a single dipole in primary motor cortex, we tested the *x*-, *y*-, and *z*-coordinates of the fitted dipoles (obtained by source analysis) against the *x*-, *y*-, and *z*-coordinates of the hand area of the primary motor cortex (obtained by meta-analysis of functional magnetic resonance imaging studies; *x* = −33, *y* = −18, and *z* = 52; Nielsen, 2003), using two-tailed Wilcoxon signed rank tests.

#### Correlation Between MEP and N100

We estimated the relationship between N100 amplitudes and interpeak MEP amplitudes across subjects for all 13 stimulation conditions using Kendall’s tau-b correlations. We likewise used Kendall’s tau-b correlations to estimate the relationship between percentage change in MEP and N100 amplitudes at the four main directions (N, E, S, W) relative to the amplitude for hot spot stimulation.

Mean N100 amplitudes at C5 in trials with high and low MEP amplitudes were tested by one-tailed Wilcoxon signed rank tests for one sample against the baseline to determine whether an N100 deflection is present also in the absence of MEPs. N100 amplitudes between trials with high and low MEPs were compared with each other with two-tailed Wilcoxon signed rank tests for two related samples.

## Results

### Topography of N100

Depicted in Figure 2, our main results demonstrate that the topographic maximum of the N100 component was ipsilateral to the stimulated hemisphere with a constant maximum posterior (approximately electrode C5) to the exact TMS application site near electrode C3. The mean N100 amplitude at electrode C5 during motor hot spot stimulation (C) was the largest (i.e., most negative) N100 amplitude (−6.8 ± 7.0 μV, mean ± standard deviation) compared with all stimulation positions. N100 amplitudes of all stimulation conditions were maximal at C5 compared with neighboring electrodes (see Supplementary Table A.1). Although Figure 2 shows small but consistent changes in the topographical maximum of the N100 in the direction of the coil position change (e.g., more posterior maximum for more posterior coil position), there were no significant topography shifts in the direction of the coil displacement. The relative amplitudes of neighboring electrodes to C5, e.g., the quotients FC3/TP7 or FC5/CP5 did not differ for coil displacements involving anterior–posterior coil shifts (all *p*s > 0.05).

### N100 Amplitudes at the Topographic Maximum (Electrode C5)

A one-way, repeated-measures Friedman’s ANOVA that tested N100 amplitude at electrode C5 as a function of the 13 stimulation sites returned a significant main effect of stimulation SITE (χ^2^ (12) = 43.6, *p* < 0.001). Bonferroni-corrected post hoc Wilcoxon tests indicated significantly larger N100 amplitudes when the coil was positioned over the motor cortex hot spot (median = −4.88 μV; *IQR* = −10.05 μV to −2.08 μV) than when the cortex was stimulated in the N condition (median = −1.15 μV, IQR = −4.60 μV to −0.46 μV; *Z* = −2.98, *p* = 0.012, *r* = −0.61), NC (median = −1.73 μV, IQR = −6.36 μV to −0.83 μV; *Z* = −2.75, *p* = 0.036, *r* = −0.56), and NE (median = −1.50 μV, IQR = −6.10 μV to −0.56 μV; Z = −2.51, *p* = 0.024, *r* = −0.51) (Figures 2, 3). The other stimulation conditions were not significantly different from the hot spot condition in terms of N100 amplitudes (*p* > 0.05).

**FIGURE 4:**
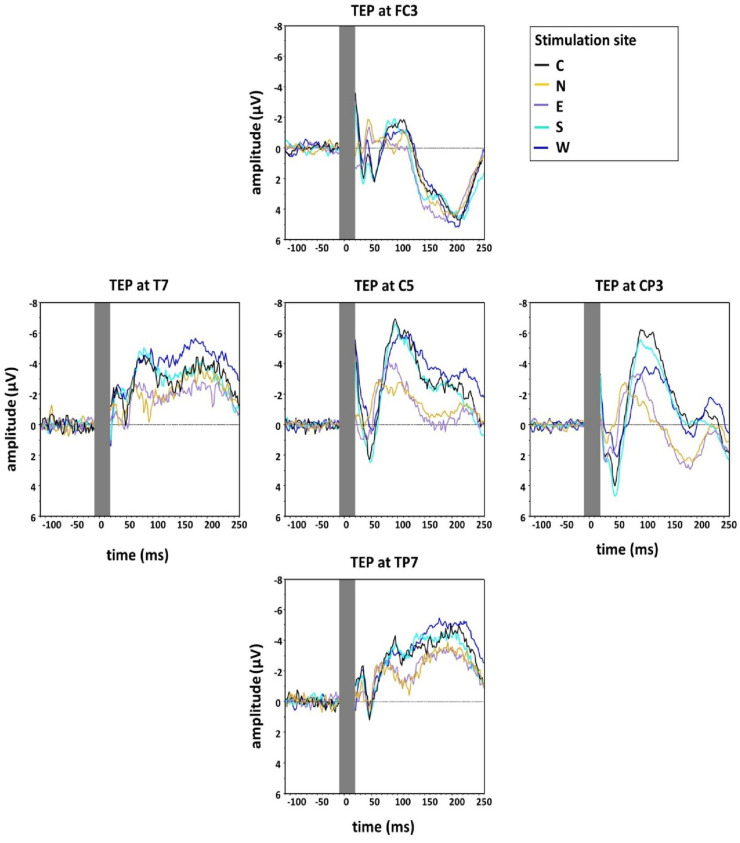
Group grand average of N100 amplitudes at the five main directions of the grid (Central, North, East, South, West) measured at five electrodes around C5 in the directions of coil displacement (C5, FC3, CP3, TP7, T7). The TMS artefact (gray bar) has been cut out.

Figure 4 shows the grand averaged TEPs for the five main stimulation conditions at the different electrodes surrounding C5 (FC3, CP3, TP7, T7), illustrating lower N100 amplitudes for the stimulation condition N.

### GFP Analysis

A one-way, repeated-measures Friedman’s ANOVA testing GFP as a function of the 13 stimulation sites revealed a significant effect of stimulation SITE (χ^2^ (12) = 21.51, *p* = 0.04). The post hoc Wilcoxon signed rank test between N (median = 2.05 μV, IQR = 1.53 μV to 3.36 μV) and hot spot condition (median = 2.37 μV, IQR = 1.97 μV to 3.97 μV) showed a one-tailed exact significant difference (*Z* = −1.88, *p* = 0.03, *r* = −0.38).

### N100 Latency

An additional explorative one-way, repeated-measures Friedman’s ANOVA testing N100 latency at electrode C5 in the time window 80–120 ms as a function of stimulation SITE (C, N, E, S, W) did not reveal a significant effect (χ^2^ (4) = 7.49, *p* = 0.11). However, as we describe above (and as depicted in Figure 3), two peaks formed in the window 80–120 ms for the N stimulation condition. N100 had an earlier rise compared with the other stimulation conditions, which was shadowed by the later second peak in the time window 80–120 ms. We accordingly conducted an additional explorative ANOVA, which again assessed the influence of stimulation SITE but this time on latencies estimated from the smaller, earlier window 80–100 ms. This ANOVA returned a main effect that trended toward significance (χ^2^ (4) = 8.84, *p* = 0.07). Post hoc comparisons suggested the N100 latency in the N stimulation condition was significantly shorter than during S stimulation (*Z* = −2.56, *p* = 0.03, *r* = −0.52). A trend was also found for the post hoc comparison between N stimulation and hot spot stimulation (*Z* = −2.41, *p* = 0.06, *r* = −0.49) (see Table 1 for descriptive statistics).

**TABLE 1:** Descriptive statistics of N100 latencies at electrode C5.

	Time window 80–120 ms		Time window 80–100 ms	
Stimulation position	Median	IQR	Median	IQR
C	96	89–110	94	86–98
N	103	92–110	87	80–90
E	93	86–100	89	86–98
S	96	86–104	96	84–98
W	96	89–101	91	84–98

*Unit of the latencies is ms; IQR = Interquartile range.*

### Source Analysis

**FIGURE 5:**
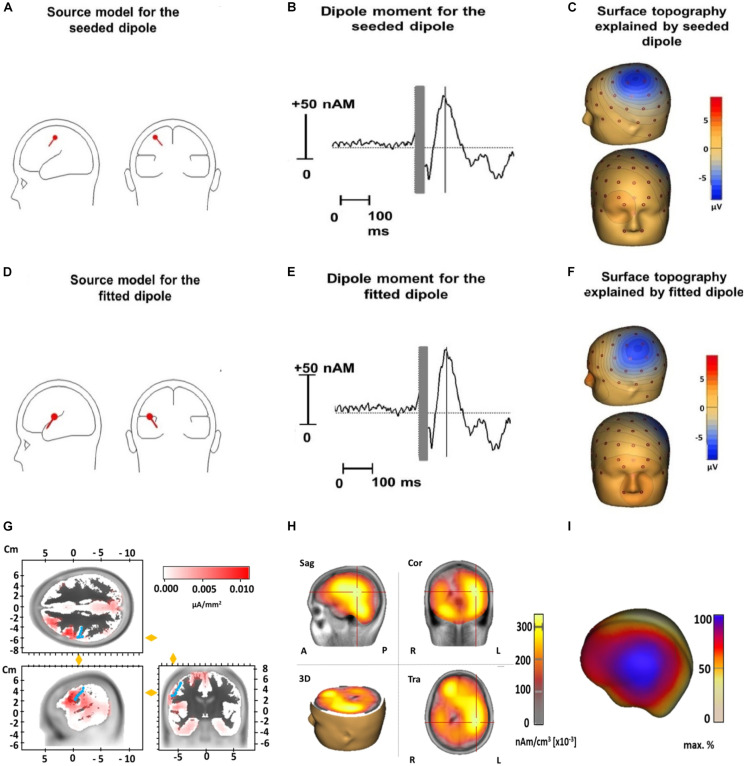
Source analysis of TMS-evoked N100 for the dipole seeded to the standard motor cortex coordinates (*x* = –33, *y* = –18, and *z* = 52) (upper row) and for the dipole fitted to our data (middle row) and LORETA results (lower row). Displayed are the dipole source localization and orientation for the seeded **(A)** and fitted dipole **(D)** and the dipole moment for the seeded **(B)** and fitted dipole **(E)** as well as the surface topography of the seeded **(C)** and fitted dipole **(F)**. Panel **(G)** illustrates LORETA results when the source space is limited to cortical areas in an averaged standard brain for the N100 time window 80–120 ms (Brain Vision Analyzer 2.2). Activation is fitted by LORETA to cortical areas in the vicinity of the precentral gyrus (marked by blue dots). Note that an exact localization without individual structural magnetic resonance images is not possible in a reliable way. Panel **(H)** shows LORETA results when the solution space is not restricted to the cortical surface in the time window 80–120 ms. Similar to Panel **(D)** it pictures a deeper activation maximum in the brain due to widespread cortical surface activation (see Panel I and Figure 2) (Sag, Sagital slices; Cor, Coronal slices; Tra, Transaxial slices; R, Right; L, Left; A, Anterior; P, Posterior). In panel **(I)** a projection of LORETA source activity on the cortical surface (BESA Research 7.1) with widespread cortical activation in the central region is displayed. This widespread topography corresponds well to the surface topography of the N100 as can be seen in Figure 2.

The median coordinates of the fitted dipole explaining the N100 TEP component were *x* = −22.1 (IQR = −38.2 to −2.80), *y* = -19.1 (IQR = −28.40 to 3.33), and *z* = 21.0 (IQR = 12.70 to 39.63). When tested against the standard motor cortex coordinates (*x* = −33, *y* = −18, and *z* = 52), there were no significant differences for the *x*- (*Z* = 1.8, *p* = 0.24) or *y*-coordinate (*Z* = 0.2, *p* = 1). However, the *z*-coordinate of the fitted dipole was significantly lower than the standard coordinate (*Z* = 3.1, *p* = 0.001), suggesting it was located significantly deeper in the brain. The fitted dipole was located directly anterior to the central sulcus (Figure 5). The fitted single dipole was able to account for 75.7% of variance in the data, whereas the seeded dipole accounted for 69.9% of variance. When a second symmetrical dipole (contralateral motor cortex, activated by transcallosal signal propagation) was introduced, the explained variance increased to 88.5% (fitted)/83.4% (seeded) in the time window 80–120 ms. These symmetrical dipoles were located at the same depth as the single dipole fitted above. No systematic residual variance remained unexplained; between 90 and 120 ms, residual variance reduced to 9%, which indicates an acceptable model fit. LORETA source analysis indicated that the deep dipole location was a consequence of widespread cortical activity, including regions outside primary motor cortex (Figure 5).

### MEP Amplitudes

**FIGURE 6:**
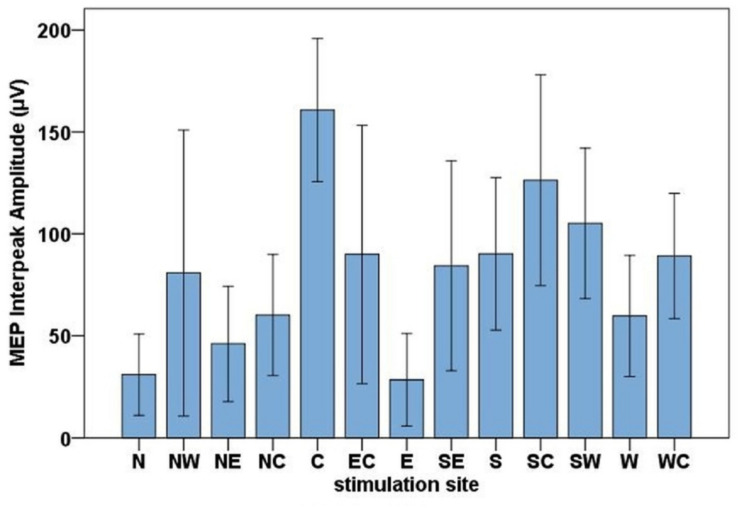
Bar chart of the mean MEP interpeak amplitudes for all stimulation sites (North, North-West, North-East, North-Central, Central, East-Central, East, South-East, South, South-Central, South-West, West, West-Central). Error bars indicate the 95 % confidence interval.

The highest MEP amplitudes were obtained during hot spot stimulation (median = 160.91 μV, IQR = 117.74 μV to 215.61 μV) (see Figure 6). A one-way, repeated-measures Friedman’s ANOVA testing MEP interpeak amplitudes as a function of stimulation SITE revealed a significant main effect (χ^2^ (12) = 58.52, *p* < 0.001; see Supplementary Table A.2 for descriptive statistics). Uncorrected two-tailed Wilcoxon signed rank tests revealed significant MEP interpeak amplitude reduction at all 12 stimulation sites compared with the hot spot condition with effect sizes (*r*) between −0.42 and −0.62. Bonferroni corrected results are displayed in Table 2.

**TABLE 2:** Two-tailed Wilcoxon signed rank tests between MEP interpeak amplitudes at the hot spot (C) and the other 12 stimulation conditions.

Comparison	Z	*p*	Effect size *r*
C to N	−3.06	0.01*	−0.62
C to S	−2.98	0.01*	−0.61
C to W	−2.90	0.01*	−0.59
C to E	−3.06	0.01*	−0.62
C to NE	−3.06	0.01*	−0.62
C to WC	−2.75	0.04*	−0.56
C to NC	−2.82	0.02*	−0.58
C to EC	−2.43	0.14	−0.50
C to SC	−2.12	0.41	−0.43
C to NW	−2.04	0.50	−0.42
C to SW	−2.35	0.19	−0.48
C to SE	−2.59	0.08	−0.53

Z, Test statistic; p, Bonferroni corrected, two-tailed exact significances; r, Effect size. * p < 0.05.

### Relationship Between Cortical Activation (N100 TEP Amplitude) and Functionally Relevant Output (Muscle Contraction, MEP Amplitude)

**FIGURE 7:**
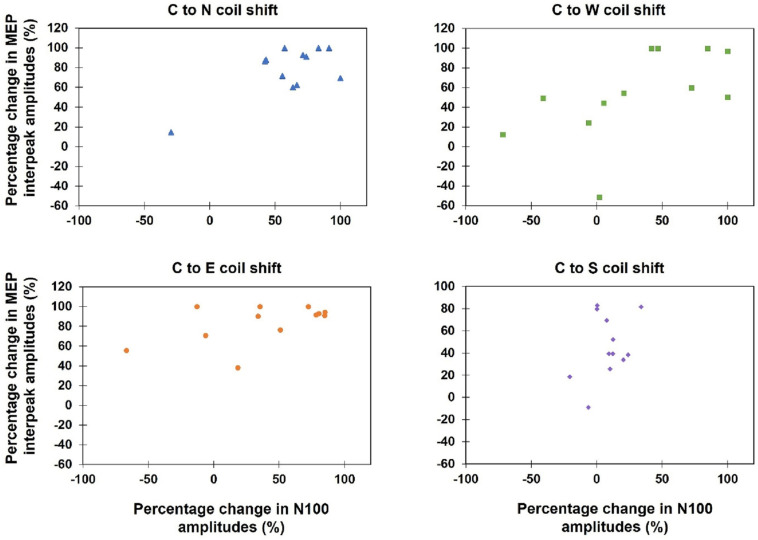
Scatterplots of the percentage change of the MEP interpeak amplitudes and N100 amplitudes, when the coil was moved away from the hot spot condition (Central) to one of the four main directions (North, East, South, West).

Kendall’s tau-b correlations revealed no significant correlations between TEPs and MEPs when the absolute amplitudes of both variables during all 13 stimulation sites were correlated (*τ*_*b*_*: p* > 0.05) or when the percentage amplitude changes between the hot spot stimulation and the four main directions were analyzed [C-W (*τ*_*b*_ = 0.50, *p* = 0.12), C-N (*τ*_*b*_ = 0.33, *p* = 0.58), C-S (*τ*_*b*_ = 0.09, *p* = 1.0), C-E (*τ*_*b*_ = 0.30, *p* = 0.76)] (see Figure 7).

N100 amplitudes differed significantly from the baseline for both trials with high (rectified amplitude > 20 μV) and low MEP amplitudes for all main stimulation conditions (C, N, E, S, W) (see Table 3).

**TABLE 3:** Two-tailed Wilcoxon signed rank tests between averaged N100 amplitudes in trials with high and low MEPs tested against the hypothetical median zero (significant deflections from baseline).

Comparison to Zero	*Z*	*P*	Effect size *r*
C- Low MEP	−3.06	0.005**	−0.62
C- High MEP	−3.06	0.005**	−0.62
S- Low MEP	−3.06	0.005**	−0.62
S- High MEP	−3.06	0.005**	−0.62
E- Low MEP	−2.93	0.01*	−0.60
E- High MEP	−2.67	0.04*	−0.45
W- Low MEP	−2.80	0.02*	−0.57
W- High MEP	−2.67	0.04*	−0.55
N- Low MEP	−2.93	0.01*	−0.60
N- High MEP	−2.93	0.01*	−0.60

Z, Test statistic; p, Bonferroni corrected, two-tailed exact significances; r, Effect size. * p < 0.05, ** p < 0.01.

However, despite TEPs being present in trials with low or absent MEP amplitudes, trials with high MEPs were accompanied by larger N100 amplitudes; before Bonferroni correction, N100 amplitudes in the high MEP conditions were significantly larger during S, W, and E stimulation and trended toward significance in the N condition (see Supplementary Table A.3 for descriptive statistics). Table 4 shows the Bonferroni corrected results.

**TABLE 4:** Two-tailed Wilcoxon signed rank tests between averaged N100 amplitudes in trials with high and low MEPs tested against each other within one stimulation condition.

Comparison	*Z*	*p*	Effect size *r*
S- Low MEP to S- High MEP	−2.98	0.005**	−0.61
C- Low MEP to C- High MEP	−2.12	0.17	−0.43
N- Low MEP to N- High MEP	−1.87	0.34	−0.38
W- Low MEP to W- High MEP	−1.99	0.25	−0.41
E- Low MEP to E- High MEP	−0.86	1	−0.18

Z, Test statistic; p, Bonferroni corrected, two-tailed exact significances; r, Effect size. ** p < 0.01.

## Discussion

The main results in this study concern coil displacement effects on N100 amplitudes and topography: TMS evoked an ipsilateral N100 TEP component with the topographic maximum located about 20 mm posterior (around electrode C5) to the stimulation site (around electrode C3). The highest mean N100 amplitudes were obtained at electrode C5 regardless of the stimulation position. However, maximum N100 amplitudes were generated during hot spot stimulation. The N100 amplitude analysis revealed significantly smaller amplitudes at electrode C5 during the N stimulation condition (anterior-medial) compared with the hot spot stimulation. The N100 topography analysis indicates a stable posterior topographical maximum at electrode C5, which did not significantly covary with small changes in stimulation site in a 4 cm^2^ area around the motor hot spot. The attenuation in N100 amplitudes during the N stimulation condition can, therefore, not be attributed to potential topography shifts.

Further analysis demonstrates significant GFP reduction during stimulation in the N compared with the hot spot condition, which points to weaker overall activation. Moreover, there was a trend toward shorter latencies for the N condition compared with both the hot spot and S stimulation conditions. Source analysis yielded an equivalent dipole located significantly deeper in the brain compared with the standard coordinates of the motor cortex. This indicates more widespread source activation than focal primary motor cortex activation. The absence of a significant correlation between MEP and TEP amplitudes might be explained by TEPs being present also in the absence of MEPs. However, we obtained hints of a subtle relationship between the two parameters. The absence of a correlation between TEP and MEP amplitude argues against TEPs being generated solely or mainly by efficient motor cortex activation.

### Relationship Between MEPs and N100

The absent correlation between MEP and N100 amplitudes is congruent with our observed differences in the effects of coil variation on both individual measures. Previous single-pulse TMS studies have also not found a correlation between MEPs and TEPs (Paus et al., 2001; Bender et al., 2005). Moreover, several studies describe TEPs for various cortices (Nikulin et al., 2003; Bender et al., 2005; Bonato et al., 2006; Rogasch et al., 2015), whereas MEPs can only be elicited in small, defined areas in primary motor cortex (Herwig et al., 2001, 2002), and TEPs can be evoked in the motor cortex independent of MEP occurrence (Bonato et al., 2006; Komssi et al., 2007).

Although MEP amplitudes seem reliant on efficient activations in corticospinal pyramidal cells (Rossini et al., 2015, 1994) TEP N100 amplitudes seem to be influenced by the number of activated GABA-B-ergic interneurons (Premoli et al., 2014). According to both ours and previous results, both processes do not seem to be strongly related: i.e., the activation of many GABA-B-ergic interneurons does not necessarily mean that a large corticospinal volley is generated in pyramidal cells and vice versa. However, on a single-trial level, we found larger N100 amplitudes in trials with high MEPs compared with low MEP trials, which replicates the finding from Fecchio et al. (2017). There seems to be a relationship between both parameters at least intraindividually if not interindividually. Previous studies speculate that stronger muscle contraction in trials with higher MEP amplitudes leads to stronger proprioceptive sensory feedback, which could alter the N100 TEP component (Fecchio et al., 2017; Petrichella et al., 2017). The significantly higher N100 amplitudes we observed in trials with high MEPs during the stimulation condition S could be due to a stronger coactivation of the primary somatosensory cortex (situated directly posterior to primary motor cortex) (Clark et al., 2010), where proprioceptive feedback and TMS-related activation might converge. However, in an earlier movement-related potential study by our group, elimination of the reafferent sensory feedback by temporary deafferentation led to a reduction of post-movement positivity in central electrodes (Thiemann et al., 2012). Therefore, it is unclear whether reafferent sensory feedback influences the negative peak of the N100 or if it is instead associated with positive deflections after movement. In sum, corticospinal and GABA-B activation seem to be separate processes, and TEPs do not solely depend on efficient motor cortex activation. There is only an indirect relationship between both parameters. Further studies should probe whether reafferent sensory feedback plays a role in shaping the N100 component.

### N100 Topography, Amplitude, and Latency

We expected the N100 topography to be both located ipsilateral to and covary with the exact coil position. Instead, we observed a stable ipsilateral, posterior topographic maximum with small, statistically insignificant topography changes in the expected directions in the group grand average. This result is consistent with earlier findings on ipsilateral topographies (Bender et al., 2005; Bonato et al., 2006; Jarczok et al., 2016), but the existing literature is inconsistent regarding the exact relationship between N100 topography and coil placement: Although some studies report N100 topographies slightly anterior to our topography at C3 (e.g., Ferreri et al., 2011) or medial central near the vertex (Du et al., 2017; Spieser et al., 2010), more widely distributed N100 topographies during primary motor cortex stimulation have also been described, often in centroparietal and parieto-occipital regions, posterior and lateral to C3 (for the left hemisphere) (Paus et al., 2001; Bonato et al., 2006; Bruckmann et al., 2012; Määttä et al., 2017; Kaarre et al., 2018). These latter results are consistent with the topography reported in our experiment. The question remains as to why we observed a stable posterior topography, i.e., topographies did not covary with stimulation position. The MEP analysis might yield information about topography characteristics because MEP amplitudes are an important indicator about how well the motor cortex is stimulated (Julkunen et al., 2009).

#### Motor Cortex Activation

MEP amplitudes were highest during hot spot stimulation and decreased in all other directions consistent with previous studies (Wassermann et al., 1992; Herwig et al., 2002; Sparing et al., 2008; Kantelhardt et al., 2010). Despite these decreases in MEP amplitude, measurable MEPs at all stimulation positions indicate activation of primary motor cortex during all stimulation positions. Considering that dipoles resulting from cortex activation are oriented perpendicular to the cortical surface (Picton et al., 1995) and that the primary motor cortex is located in the crown and posterior wall of the precentral gyrus (bordering the central sulcus) (Sanes et al., 1995; Amunts et al., 1997), the posterior topography may be explained by a primary motor cortex dipole (Bruckmann et al., 2012).

Ahdab et al. (2016) demonstrate that the hand motor hot spot is located over the central sulcus in 45% of a sample of healthy subjects, mostly at the position of the hand knob in the primary motor cortex. In most of the other cases however, they located the hot spot more anteriorly in the precentral or middle frontal gyrus. We also observed variability in hot spot locations across subjects (see Figure 1). Although most of the hot spots were located near electrode C3 or slightly medial, three participants exhibited slightly more anterior hot spots near electrode FC3. As previously stated, the position of electrode C3 (in the 10–20 electrode system) is associated with the hot spot location for the FDI muscle (Rogasch et al., 2013; Holmes and Tamè, 2019). Although three participants featured slightly more anterior hot spots, on average, the focus of the cortical activation in our sample was likely in the primary motor cortex around the posterior wall of the precentral gyrus, bordering the central sulcus. Thus, a posterior topography might be explained by consistent primary motor cortex activation, independent of the exact stimulation position and the anatomical characteristics of the primary motor cortex.

#### Activation of Surrounding Cortical Areas

On the one hand, topographies consistently posterior to the stimulation site may be caused by consistent activation of primary motor cortex regions that vary in size. On the other hand, the equivalent dipole we observed at a significantly deeper location to the standard motor cortex coordinates, in addition to the widespread cortical activity revealed in the LORETA source analysis, are inconsistent with exclusive activation of the primary motor cortex. Although other studies demonstrate an equivalent dipole near the cortical surface (Helfrich et al., 2012; Määttä et al., 2017), Bruckmann et al. (2012) identify a more deeply localized dipole, similar to our findings. We attribute the deeper location of the dipole to a source-analytic solution that explains the broad surface activity around the TMS stimulation site by concentrating the activity into a deeper localized equivalent dipole (Figure 5). Because primary motor cortex is closer to the scalp, a sole activation of primary motor cortex would lead to a less widespread topography. Thus, contrary to our expectations, the N100’s equivalent dipole location likely indicates activations in a larger cortical area around the hot spot. Even though the fitted dipole only accounted for approximately 6% more variance than the seeded dipole, this small difference in variance nonetheless seems relevant given that the fitted dipole was located significantly deeper than the seeded dipole. Thus, although these results are limited by the inverse problem of source analysis and the limited spatial resolution of EEG, the deeper located dipole indicates activations in a broader cortical area around the primary motor cortex.

#### Differences in Cortical Excitability

Our results concerning the N100 amplitude and latency as well as GFP further support the conclusion that TMS stimulated regions beyond the primary motor cortex. Exclusive activations within the primary motor cortex should yield either both N100 and GFP amplitude reduction during stimulation away from the hot spot (similar to the MEP amplitude changes) or there should be no changes in TEP amplitudes, similar to the study from de Goede et al. (2018). These authors observed no differences in TEP amplitudes across nine stimulation positions 2 or 5 mm around the hot spot within a latency of 100 ms. Considering that primary motor cortex extends medially (approximately 43.6 mm) and laterally (approximately 22.7 mm) from the motor hot spot (Lotze et al., 2000) but to a lesser extent anteriorly and posteriorly (width of precentral gyrus is approx. 16 ± 3.6 mm) (Ebeling et al., 1986), de Goede et al. (2018) may have stimulated only the primary motor cortex and, therefore, measured similar TEP amplitudes. We hypothesize that, even with larger (5–10 mm) coil deviations around the hot spot, we also stimulated mainly the primary motor cortex during medial (E) and lateral (W) stimulation due to the medial and lateral primary motor cortex expansion. This would explain similar N100 amplitudes during E, W, and hot spot stimulation.

However, given the small anterior–posterior width of the primary motor cortex, we cannot assume that this cortical area was stimulated exclusively on the anterior–posterior axis (N and S), considering the greater coil deviation in our study. Our source analysis results suggest that TMS evoked the N100 component in a wider cortical area. Therefore, different effects between the N and other locations could be explained by different properties of the targeted cortical areas. As the premotor cortex is located anterior to the primary motor cortex (Clark et al., 2010), we might have partially stimulated the premotor cortex during N stimulation as well, at least to a stronger extent than during motor hot spot stimulation. Fecchio et al. (2017) demonstrate that stimulating the primary motor cortex leads to significantly larger TEP amplitudes than stimulating the anteriorly located premotor cortex, which suggest a higher local cortical excitability for primary motor cortex. The findings by Fecchio et al. (2017) are in line with other research suggesting that the primary motor cortex is generally more excitable than prefrontal cortical areas (Kähkönen et al., 2005, 2004) and produces higher N100 amplitudes (Lioumis et al., 2009).

Latency differences between motor areas are also reported in previous experiments: TEP components P30 and N45 occurred earlier during premotor cortex than during primary motor cortex stimulation (Van Der Werf and Paus, 2006). A descriptive analysis of global mean field amplitudes of later peaks (around 100 ms) reveals shorter mean latencies during stimulation of the supplementary motor area and dorsal premotor areas compared with stimulation of the primary motor cortex (Salo et al., 2018). These findings are only descriptive in nature, but they describe shorter TEP latencies during stimulation of anterior motor areas. The shorter N100 latencies found in our study for the N condition further support the conclusion of premotor cortex stimulation.

As previously implied, we might not have exclusively stimulated primary motor cortex in the S condition either. Because the somatosensory hand representation is only ∼15 to ∼5 mm posterior lateral to the hand motor cortex hot spot (Holmes and Tamè, 2019), we might have stimulated the somatosensory cortex to a larger extent in the S than in the motor hot spot stimulation condition. Nonetheless, we found similar N100 amplitudes in the S and hot spot conditions, which might be due to similar excitability in both cortices. To our knowledge, no (TMS-EEG) study has compared the excitability of both cortices. On the basis of previous reports of higher N100 amplitudes in sensorimotor areas compared with frontal areas (Kähkönen et al., 2005, 2004; Lioumis et al., 2009), we speculate that more posterior cortices may have similar excitabilities and N100 amplitudes (i.e., primary motor cortex and somatosensory cortex), whereas more anterior cortices may be less excitable (i.e., premotor cortex). This would explain the anterior–posterior differences in our study.

#### GABA-B Neurotransmission

Why anterior cortical areas show lower excitability and shortened N100 latencies cannot be answered conclusively with the present data. However, neurotransmitter receptor density varies even within the sensorimotor cortex (Zilles et al., 2002, 1995). Hence, a possible hypothesis is that cytoarchitectural differences between cortices provide the basis for different N100 components. Because GABA-B neurotransmission plays an important role in forming the N100 component (Premoli et al., 2014) GABA-B receptor density in distinctive cortical areas might explain differences in N100 amplitude. Although one study observed no difference in GABA-B receptor density between primary and premotor cortex (Zilles and Palomero-Gallagher, 2017), the anterior primary motor cortex has fewer GABA-B receptors than the posterior primary motor cortex (Zilles et al., 2002). Additionally, the somatosensory cortex, posterior to the primary motor cortex, shows a higher density of GABAergic receptors than the primary motor cortex (Zilles et al., 1995). These findings support our observed lower N100 amplitudes following more anterior stimulation and higher amplitudes following more posterior stimulation. However, amplitudes in the S conditions were similar to rather than higher than amplitudes in the hot spot condition. Although this discrepancy cannot be resolved conclusively in our study, it could be due to partial and/or inefficient somatosensory cortex stimulation because the coil orientation is optimized for primary motor cortex stimulation on the basis of motor output (MEPs). Sole and/or efficient somatosensory cortex stimulation might lead to higher N100 amplitudes compared with primary motor cortex stimulation, but this hypothesis needs to be evaluated in future studies. In addition, there are indications that GABA-B postsynaptic potential latencies lengthen with an increasing number of axons converging on the postsynaptic cell (Thomson and Destexhe, 1999). Therefore, levels of GABA-B receptor density varying between cortical areas might explain differences in N100 amplitudes and variations in N100 latencies. This could be the reason that mean latencies were shortest in the N condition and longest during S stimulation. Consequently, the N100 latencies should differ between cortices with diverging cytoarchitectures (Zilles et al., 2002). Instead, inconsistent results have been found: In some studies, no differences in N100 latencies were detected between different cortical areas, such as the dorsolateral prefrontal cortex (DLPFC) and primary motor cortex (Lioumis et al., 2009) or between prefrontal, motor, primary auditory cortices; the vertex; and the posterior cerebellum (Du et al., 2017). However, other studies point to longer latencies in the posterior cortex (Rosanova et al., 2009; Herring et al., 2015; Samaha et al., 2017) compared with anterior cortices (Lioumis et al., 2009). Although topographical variation in N100 amplitudes and possibly latencies might be explained by regional differences in cortical excitability or GABA-B-ergic neurotransmission in the respective cortical areas, the relationship between GABA-B receptor density and N100 amplitudes and latency is inconclusive and needs to be evaluated systematically in future experiments. It is also important to consider that N100 amplitude may not only reflect GABA-B activity, but a local balance of glutamate and GABA (Du et al., 2018). Therefore, GABA-B receptor distribution is only one of several possible factors that could influence characteristics of the N100.

In conclusion, TMS seems to depolarize a large cortical area around the actual target site (as the source analysis indicates) involving not only primary motor cortex, but also adjoining cortical areas. This may result in smaller N100 and GFP amplitudes as well as shorter N100 latencies during anterior stimulation. MEPs evoked during stimulation anterior to the motor hot spot (smallest N100 amplitude) illustrate that the primary motor cortex was still activated to some extent in all stimulation conditions. Consistent partial primary motor cortex activation for all coil positions combined with the generation of high N100 amplitudes in the primary motor cortex might have contributed to a rather stable N100 topography.

Nonetheless, our results would possibly change with increased spatial displacement of the coil from the primary motor cortex. The spatial change in coil positions in our study with a maximum inter-site distance of 20 mm (between N–S or W–E) may have been too small to drive significant covariation in topographies. Greater coil displacement might reduce primary motor cortex coactivation and change N100 topographies such that maxima fall over the stimulation site. Further studies should, therefore, choose larger distances between the coil positions to identify the respective contributions of anatomical characteristics in the primary motor cortex and local excitability in other cortical areas on N100 topographies.

### Limitations

A limitation of our study was that we did not minimize the auditory response to the coil click by applying auditory masking. During TMS-EEG recording, the clicking noise leads to auditory evoked potentials (AEPs), which consist of an N1–P2 complex—a negative peak after 100 ms and a positive peak varying between 180 and 200 ms (Nikouline et al., 1999; Rogasch et al., 2014; ter Braack et al., 2015; Conde et al., 2019). In particular, the negative AEP peak around 100 ms might distort the TMS-evoked N100 component (Nikouline et al., 1999; Rogasch et al., 2014; ter Braack et al., 2015). Therefore, it is likely that the measured N100 amplitude in our study comprises not only genuine cortical activity due to the TMS pulse, but also peripherally evoked AEPs. However, AEPs have little impact on N100 amplitudes (Du et al., 2017), and the topographies of the N100 and P200 in our study do not support the assumption that the transcranially evoked N100 was strongly distorted by AEPs. The AEP-related P200 component is generally not lateralized and has its topographical maximum in fronto-central regions at Cz and Fz (Goff et al., 1977; Hine and Debener, 2007), and the AEP-related N100 component either peaks at the vertex (Rogasch et al., 2014; Conde et al., 2019) or features shorter latencies and higher amplitudes in the contralateral hemisphere during monaural acoustic stimulation (McCallum and Curry, 1980; Hine and Debener, 2007). A clearly lateralized ipsilateral N100 topography is inconsistent with the component reflecting exclusively peripherally evoked potentials (Conde et al., 2019). In Figure 2, we demonstrated that our N100 component is clearly ipsilateral and the P200 component is constantly localized in fronto-central regions (see Supplementary Figure B.1). Although the existence of the fronto-central P200 component illustrates that the coil click generated AEPs, the clearly ipsilateral, rather than contralateral, topography of the N100 cannot be explained by peripherally evoked potentials and is more likely to be genuine cortical activation due to the TMS pulse. Finally, we checked the time course in channels P9/P10 (not shown), in which the “positive pole” of an auditory cortex dipole would appear and compared them with the time course of fronto-central electrodes. We did not observe any evidence that our results were distorted by AEPs in the N100 latency range (shorter latency, shorter peak duration, contralateral maximum of putative AEPs).

This study did not use a neuronavigation system to track the exact coil movement. Frameless neuronavigation makes it possible to guide the movement of the TMS coil based on brain imaging data and helps to locate and maintain precise stimulation sites (Herwig et al., 2002; Schönfeldt-Lecuona et al., 2005). Despite these positive aspects, neuronavigation is a complex procedure that significantly prolongs experiment duration for participants (Julkunen et al., 2009). Instead, we repeatedly ensured (visually) that the coil was positioned tangentially at a 45° angle from the midsagittal line and reliably at the exact determined coil location. The MEP assessment was explicitly used to externally verify correct coil positioning. Therefore, even though we did not use neuronavigation, we believe that our results provide a deeper insight into the relationship between coil positioning and the basic principles of the N100. Nonetheless, our results need to be replicated by further studies with neuronavigation.

In this study, we investigated the effects of coil position in a small area around the dominant left motor cortex of right-handed participants. Future studies need to replicate our study while applying TMS at larger distances between the stimulation points, map different cortical (nonmotor) areas, add auditory masking, and use additional neuronavigation to record the exact stimulated cortical area.

## Conclusion

In summary, we demonstrate that TMS over the primary motor cortex leads to an ipsilateral N100 topography, which might be influenced and generated by both the anatomical properties of the primary motor cortex and the local excitability of the surrounding cortical areas. The N100 might, therefore, be suitable for illustrating differences in local excitability/inhibition, leading to characteristic differences in amplitude and latency during cortical mapping. This cortical activation, most likely related to GABA-B-ergic neurotransmission, is largely independent of MEP generation in the motor cortex, which depends on pyramidal cell activation and corticospinal volley. Therefore, both parameters—MEPs and the N100—provide complementary information on cortical excitability. Nonetheless, these results need to be replicated in neuronavigated studies.

## Data Availability Statement

The datasets presented in this article are not readily available because online data sharing was not common practice at the time the study was designed (2017). Therefore, the participants and the local ethics committee were assured that the data would remain confidential. Requests to access the datasets should be directed to LB, lea.biermann@uk-koeln.de.

## Ethics Statement

The studies involving human participants were reviewed and approved by Ethics Committee of the Faculty of Medicine, University of Cologne, Germany. The patients/participants provided their written informed consent to participate in this study.

## Author Contributions

SB contributed to both the conception and design of the study, provided supervision for data analysis and resources for the study, and edited the manuscript. DR organized and conducted the investigation. DR and LB performed the data analysis and wrote the first draft of the manuscript together. LB performed the statistical analysis and visualized the results. TJ provided supervision, contributed to data analysis and to writing and editing the manuscript. All authors read and approved the submitted version.

## Conflict of Interest

The authors declare that the research was conducted in the absence of any commercial or financial relationships that could be construed as a potential conflict of interest.

## Footnotes

1 http://www.clinicalresearcher.org/software.htm
