# Corrigendum: Local Differences in Cortical Excitability – a Systematic Mapping Study of the TMS-Evoked N100 Component

**DOI:** 10.3389/fnins.2021.709605

**Published:** 2021-06-10

**Authors:** Daniela Roos, Lea Biermann, Tomasz A. Jarczok, Stephan Bender

**Affiliations:** Department of Child and Adolescent Psychiatry, Psychosomatics, and Psychotherapy, Faculty of Medicine and University Hospital Cologne, University of Cologne, Cologne, Germany

**Keywords:** N100, TMS-EEG study, motor cortex mapping, cortical excitability, relationship N100 and MEP

In the original article, we neglected to include the funder Marga und Walter Boll-Stiftung, grant no.: 210-04.00-16 to Stephan Bender. The corrected funding statement is shown below.

## Funding

This research was funded by the Deutsche Forschungsgemeinschaft (DFG, German Research Foundation) – Project-ID 431549029 – SFB 1451 and Marga und Walter Boll-Stiftung, grant no.: 210-04.00-16.

The authors apologize for this error and state that this does not change the scientific conclusions of the article in any way. The original article has been updated.

