# Supplementary material for: Local Differences in Cortical Excitability – A Systematic Mapping Study of the TMS-Evoked N100 Component
Source: Front Neurosci. 2021 Feb 25;15:623692. doi: 10.3389/fnins.2021.623692 (PMC7959732; doi:10.3389/fnins.2021.623692)
Supplement: Supplementary file 1 [file Data_Sheet_1.PDF]

Supplementary Material

1 Supplementary Tables A

Table A.1. *Descriptive statistics of N100 amplitudes at all electrodes of interest during all 13 stimulation sites*

Electrode	Stimulation site	*N*	*M*	*SD*	*Median*	*IQR*
	N	12	-2.98	4.32	-1.15	-4.60 – -0.46
	NC	12	-4.08	5.74	-1.73	-6.36 – -0.83
	C	12	-6.76 *	7.02	-4.88 *	-10.05 – -2.08
	SC	12	-6.47	7.04	-3.38	-11.23 – -1.77
C5	S	12	-6.32	6.63	-3.75	-9.62 – -1.73
	SW	12	-6.52	8.97	-2.85	-10.06 – -1.79
	W	12	-6.43	12.52	-2.22	-5.80 – -0.85
	WC	12	-5.11	6.76	-2.49	-6.51 – -2.23
	NW	12	-5.05	7.70	-2.13	-7.08 – -1.13
	NE	12	-3.49	4.22	-1.50	-6.10 – - 0.56
	E	12	-3.92	3.99	-2.39	-7.33 – -0.68
	EC	12	-4.99	5.30	-2.63	-8.58 – -1.03
	SE	12	-5.65	4.21	-4.71	-8.50 – -2.32
	N	12	-0.88	1.17	-0.56	-1.07 – -0.11
	NC	12	-0.86	1.15	-0.40	-1.43 – 0.00

	C	12	-1.98	2.86	-1.13	-2.93 – 0.00
	SC	12	-1.53	2.01	-0.39	-3.33 – 0.00
	S	12	-1.82	2.12	-1.28	-2.82 – 0.00
	SW	12	-1.40	3.44	-0.11	-0.84 – 0.00
FC3	W	12	-2.20	4.84	0.00	-2.04 – 0.00
	WC	12	-1.16	1.82	0.00	-1.61 – 0.00
	NW	12	-1.35	2.49	-0.54	-1.40 – 0.00
	NE	12	-1.36	1.51	-1.20	-2.68 – 0.00
	E	12	-0.75	1.13	-0.10	-1.44 – 0.00
	EC	12	-1.09	1.27	-0.26	-2.42 – 0.00
	SE	12	-0.98	1.29	-0.50	-1.48 – 0.00
	N	12	-2.05	3.34	-0.49	-3.57 – 0.00
	NC	12	-3.55	5.36	-1.43	-5.40 – -0.19
	C	12	-6.57	7.28	-4.50	-9.18 – -2.15
	SC	12	-5.63	6.62	-3.19	-9.51 – -1.45
CP3	S	12	-5.81	7.04	-2.61	-9.02 – -1.50
	SW	12	-5.33	8.22	-1.95	-7.11 – -1.54
	W	12	-5.10	11.09	-1.22	-3.97 –0.13
	WC	12	-4.70	6.46	-2.23	-5.73 – -0.75
	NW	12	-4.46	7.18	-3.33	-4.02 – -0.07

	NE	12	-2.51	3.50	-0.88	-5.44 – -0.01
	E	12	-3.52	4.32	-1.80	-6.42 – 0.00
	EC	12	-4.93	5.22	-3.08	-7.89 – -0.74
	SE	12	-4.84	4.01	-3.64	-7.74 – -1.74
TP7	N	12	-1.97	2.15	-1.59	-4.03 – 0.00
	NC	12	-2.98	3.07	-1.44	-4.58 – -1.24
	C	12	-3.92	3.30	-2.93	-7.31 – -0.82
	SC	12	-4.21	3.98	-2.43	-7.73 – -1.48
	S	12	-3.72	3.28	-2.73	-6.48 – -0.50
	SW	12	-3.73	3.35	-2.69	-7.29 – -1.24
	W	12	-3.91	4.63	-3.11	-6.01 – -0.46
	WC	12	-3.22	3.23	-1.96	-6.32 – -0.66
	NW	12	-2.76	3.20	-2.33	-4.36 – -0.14
	NE	12	-2.12	2.16	-1.56	-3.74 – -0.23
	E	12	-2.51	2.18	-2.23	-4.34 – -0.41
	EC	12	-3.28	2.67	-2.94	-5.60 – -1.48
	SE	12	-3.65	2.69	-3.10	-6.12 – -1.09
	N	12	-2.07	2.77	-1.37	-3.13 – 0.00
	NC	12	-2.72	3.66	-1.43	-3.45 – -0.25
	C	12	-4.21	4.76	-2.42	-7.36 – -0.47
	SC	12	-4.96	6.24	-2.56	-5.88 – -1.35

	S	12	-4.61	6.48	-2.10	-5.93 – -0.67
	SW	12	-5.46	7.75	-2.38	-6.25 – -0.74
T7	W	12	-4.69	5.99	-2.74	-6.19 – -0.96
	WC	12	-3.31	4.09	-1.72	-4.99 – -0.84
	NW	12	-2.91	4.31	-0.71	-3.62 – -0.23
	NE	12	-2.38	3.20	-1.45	-3.64 – -0.04
	E	12	-2.39	2.51	-1.42	-4.46 – -0.64
	EC	12	-2.88	3.16	-1.86	-4.11 – -0.96
	SE	12	-4.26	3.32	-3.04	-6.60 – -1.91
	N	12	-2.67	4.86	-0.62	-1.97 – -0.16
	NC	12	-3.08	5.46	-1.38	-2.46 – -0.21
	C	12	-4.40	5.63	-2.67	-4.99 – -0.30
	SC	12	-4.43	6.92	-2.66	-4.32 – -0.02
	S	12	-4.46	6.80	-1.57	-6.11 – -0.05
FC5	SW	12	-5.10	8.58	-1.64	-3.32 – -0.34
	W	12	-4.97	9.29	-0.64	-5.22 – 0.00
	WC	12	-3.50	5.86	-0.97	-4.60 – -0.13
	NW	12	-3.84	6.39	-1.63	-3.33 – 0.00
	NE	12	-2.95	5.10	-0.80	-3.60 – -0.11
	E	12	-1.89	2.78	-0.70	-3.06 – -0.07

	EC	12	-3.02	4.54	-1.62	-3.19 – -0.15
	SE	12	-3.34	3.76	-2.89	-4.07 – -1.01
	N	12	-2.13	3.14	-0.94	-2.99 – 0.00
	NC	12	-3.58	5.40	-1.60	-4.56 – -0.19
	C	12	-6.28	6.24	-4.51	-7.72 – -2.18
	SC	12	-5.70	6.66	-3.38	-7.68 – -1.71
	S	12	-5.66	6.57	-3.41	-8.74 – -1.18
	SW	12	-5.41	7.50	-2.88	-5.62 – -1.20
CP5	W	12	-5.30	10.67	-1.65	-3.84 – -0.25
	WC	12	-4.56	6.16	-2.22	-4.76 – -0.89
	NW	12	-4.19	6.85	-2.53	-3.40 – -0.38
	NE	12	-2.46	3.24	-1.36	-4.25 – -0.20
	E	12	-3.58	3.80	-2.20	-6.69 – -0.54
	EC	12	-4.64	4.79	-3.23	-7.67 – -0.43
	SE	12	-5.04	4.01	-4.41	-6.92 – -2.36

Note: Unit of the amplitudes is µV; *N* = Sample size; *M* = Arithmetic mean; *SD* = Standard Deviation; *IQR* = interquartile range between 25 % and 75 % quartile
* marks the highest arithmetic mean and median.

Table A.2. *Descriptive statistics of MEP Interpeaks per stimulation position*

Stimulation site	*N*	*M*	*SD*	*Median*	*IQR*
MEPInterpeak C	12	160.77*	55.40	160.91*	117.74 – 215.61
MEPInterpeak N	12	30.94	31.33	22.36	2.59 – 52.52
MEPInterpeak S	12	90.18	58.93	98.39	28.30 – 142.06
MEPInterpeak W	12	59.70	46.82	67.11	1.33 – 99.65
MEPInterpeak E	12	28.49	35.61	16.08	2.65 – 38.16
MEPInterpeak SE	12	84.31	81.09	68.41	14.64 – 104.42
MEPInterpeak NE	12	46.06	44.35	31.68	13.70 – 76.16
MEPInterpeak SW	12	105.17	58.12	95.83	56.50 – 131.99
MEPInterpeak WC	12	89.15	48.35	89.22	52.89 – 110.13
MEPInterpeak NW	12	80.84	110.38	46.97	7.64 – 108.80
MEPInterpeak NC	12	60.14	46.83	44.53	26.28 – 98.57
MEPInterpeak SC	12	126.30	81.45	113.83	46.19 – 188.22
MEPInterpeak OC	12	89.97	99.74	54.75	14.78 – 136.18

Note: Unit of the amplitudes is µV; *N* = Sample size; *M* = Arithmetic mean; *SD* = Standard Deviation; *IQR* = interquartile range between 25 % and 75 % quartile
* marks the highest arithmetic mean and median.

Table A.3. *Descriptive statistics of N100 amplitudes at electrode C5 in trials with high and low MEPs*

Simulation site		*N*	*M*	*SD*	*Median*	*IQR*
S	High MEP	12	-8.50*	9.41	-5.29*	-9.75 – -2.94
	Low MEP	12	-5.78	6.29	-3.68	-8.81 – -1.44
W	High MEP	12	-7.26	13.29	-2.91	-7.99 – -0.32
	Low MEP	12	-6.08	11.60	-2.25	-5.99 – -0.74
C	High MEP	12	-7.21	7.63	-4.83	-10.24 – -2.13
	Low MEP	12	-5.60	6.68	-3.63	-8.95 – -1.13
E	High MEP	12	-4.04	4.64	-2.00	-7.76 – -0.12
	Low MEP	12	-3.58	3.82	-1.98	-7.16 – -0.81
N	High MEP	12	-3.99	5.01	-2.64	-6.63 – -0.26
	Low MEP	12	-2.93	4.44	-1.17	-3.86 – -0.82

Note: Unit of the amplitudes is µV; *N* = Sample size; *M* = Arithmetic mean; *SD* = Standard Deviation; *IQR* = interquartile range between the 25 % and 75 % quartile
* marks the highest arithmetic mean and median.

2 Supplementary Figures B

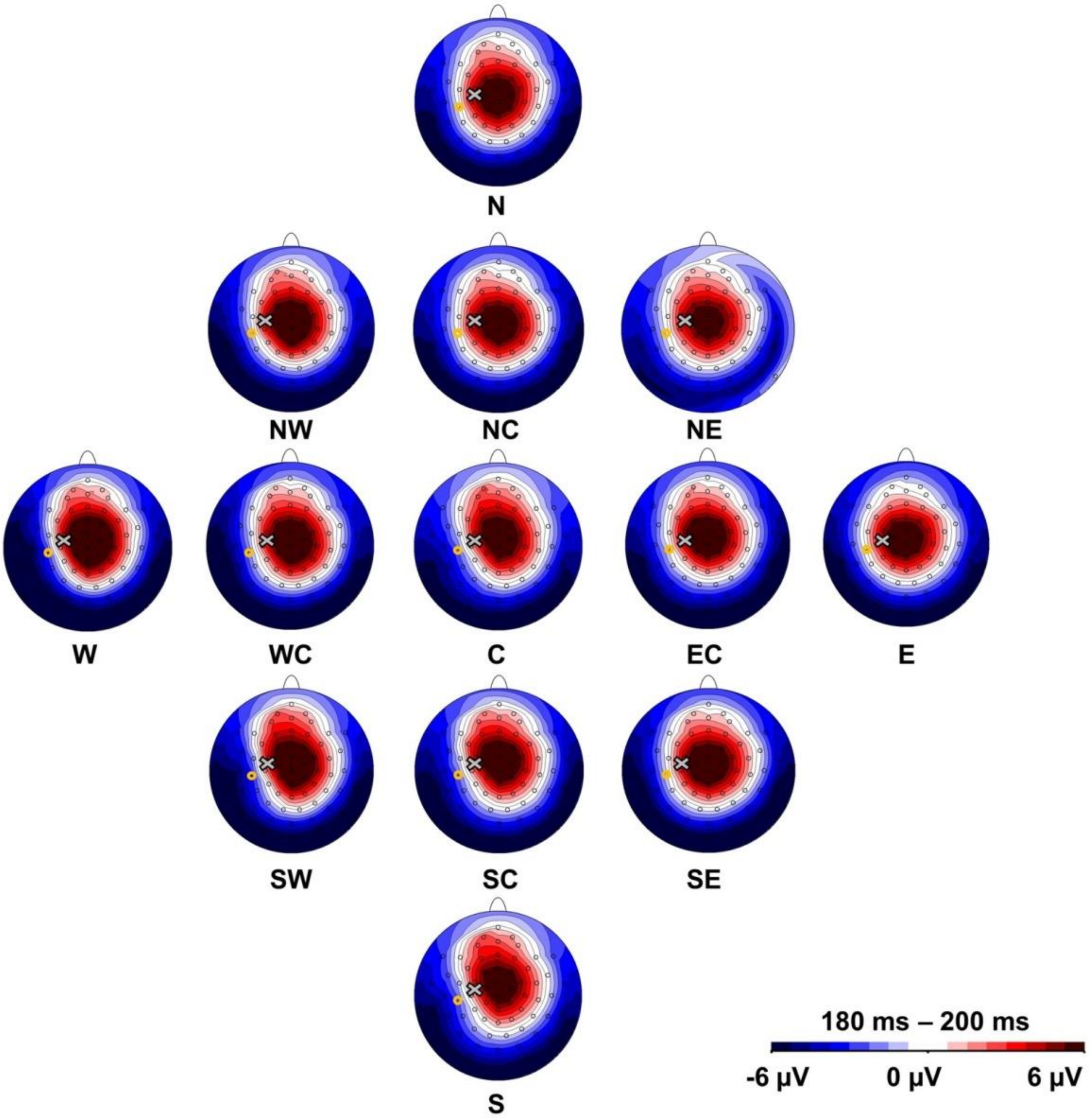

Supplementary Figure B.1. Topography of the P200 component for all stimulation conditions (**N**orth, **N**orth-**W**est, **N**orth-**E**ast, **N**orth-**C**entral, **C**entral, **E**ast-**C**entral, **E**ast, **S**outh-**E**ast, **S**outh, **S**outh-**C**entral, **S**outh-**W**est, **W**est, **W**est-**C**entral). Yellow circles mark the position of electrode C5 and the cross marks the approximate TMS-stimulation site during hotspot stimulation (group average). The view is from the top of the head with the nose pointing upward.
